# Subjective Sleep Quality as a Possible Mediator in the Relationship between Personality Traits and Depressive Symptoms in Middle-Aged Adults

**DOI:** 10.1371/journal.pone.0157238

**Published:** 2016-06-10

**Authors:** Vivian Huang, Katlyn Peck, Sasha Mallya, Sonia J. Lupien, Alexandra J. Fiocco

**Affiliations:** 1 Department of Psychology, Ryerson University, Toronto, Ontario, Canada; 2 Department of Psychiatry, Université de Montréal, Montreal, Quebec, Canada; University of Illinois at Chicago College of Medicine, UNITED STATES

## Abstract

This study explored the mediating role of sleep in the relationship between personality traits and depressive symptoms in a group of community-dwelling men and women (*M*_age_ = 57.92, *SD* = 4.00). Participants completed the short form NEO Five Factor Inventory (NEO-FFI), Pittsburgh Sleep Quality Index (PSQI), and the Center for Epidemiologic Studies Depression Scale (CES-D). High neuroticism and low conscientiousness was associated with poor sleep, as well as greater depressive symptom severity. Partial indirect mediation effects were found between personality traits (i.e., neuroticism and conscientiousness) and depressive symptoms through self-report sleep measures. An alternative model was also explored, entering depression as the mediator; however a smaller portion of the variance was explained by this model, compared with the hypothesized model. The current study provides preliminary information regarding the mechanisms that influence the relationship between personality traits, sleep, and depression among a group of community-dwelling middle-aged adults. Implications and future directions are discussed.

## Introduction

Depression is the leading cause of disability worldwide, and is a major contributor to the overall global burden of disease [1]. Regardless of severity, depression is associated with poor physical function, greater healthcare utilization, morbidity, and increased mortality [2,3]. Unfortunately, one-fourth of individuals with depression are left undiagnosed [4] and fewer than half receive treatment [5, 6], resulting in accelerated deterioration in physical health and overall quality of life. Considering the widespread implications of depression, it is imperative to elucidate underlying mechanisms associated with depressive symptoms.

Among the various risk factors that have been identified for depression, including chronic health conditions and previous history of depression [7], sleep disturbance has received increasing attention over the last decade. The relationship between sleep and depression is bi-directional, regardless of age [8, 9]. While sleep quality is a substantial factor contributing to a healthy lifestyle, sleep disturbances increase the risk for depression, as evidenced by symptoms including increased guilt and the inability to concentrate [10]. Importantly, mounting evidence implicates sleep disturbances as a precursor to the onset of depression, rather than merely a symptom [11, 12]. Conversely, depression is associated with difficulties with initiating or maintaining sleep, resulting in insomnia or hypersomnia, which further contribute to the persistence of low mood [13, 14]. Overall, previous studies suggest that sleep disturbance may serve as one of the risk factors for onset, persistence, and recurrence of depression [11, 15].

Although sleep and depression are closely linked, few studies have evaluated the effect of individual difference factors that may contribute to this relationship. A factor that may serve as a measure of individual differences in the sleep-depression association is personality. Personality traits provide a stable measure of individual differences in emotional and cognitive processes, as well as behavioural tendencies [16]. Given that emotions, cognitions and behaviours are significant factors that underlie depression and sleep problems [17], it is likely that personality may play a role in the interplay between sleep and depression.

The Five Factor Model is one of the most established and widely used personality taxonomies [18]. The model includes neuroticism, extraversion, openness, agreeableness, and conscientiousness [19, 18, 20]. Neuroticism characterizes individuals who have a tendency to worry and report negative affect [21]; Extraversion characterizes an individual as sociable, cheerful, and assertive; Openness to Experience characterizes individuals who are creative and open-minded; Agreeableness characterizes individuals who are trusting, generally compliant, and sympathetic, and Conscientiousness characterizes individuals who are competent, well-organized, and self-disciplined [22].

Previous studies suggest that higher self-reported neuroticism is associated with sleep disturbances and poor sleep quality [23–25], and variability in sleep duration [26, 27]. A recent population-based study examined the association between the Five Factor personality traits and self-reported sleep quality in two different young adult cohorts [25]. Findings revealed that high extraversion, agreeableness, and conscientiousness were associated with better sleep, whereas high neuroticism was associated with poor sleep. Of the five traits, openness was not associated with sleep [25]. McHugh et al. [28], examined the personality correlates of sleep quality, namely extraversion and neuroticism, in a sample of community-dwelling older adults. While no relationship was reported for extraversion, high neuroticism was associated with self-identified poor sleepers.

Notably, the heightened vulnerability to poor sleep quality among individuals who are high in neuroticism may be influenced by their level of conscientiousness. For instance, Williams and Moroz [29] examined the association between levels of neuroticism and conscientiousness on sleep quality and other health-related outcomes in a group of first year college students. Results at 2-months follow up demonstrated that neuroticism predicted poor overall subjective sleep quality, while conscientiousness was associated with enhanced sleep quality [29]. Individuals scoring high on neuroticism and low on conscientiousness were found to experience the greatest amount of dysfunction in relation to perceived sleep loss, and in turn, the consequences of poor sleep in this group was associated with experience of concurrent depressive symptoms [29]. Additional research supports that the combination of high neuroticism and low conscientiousness is particularly detrimental, since it is associated with poor overall sleep quality relative to all other personality traits [30].

Although research does provide support for strong associations between single personality factors as predictors of health outcomes, increasing evidence suggests the interactive effect of multiple traits such as neuroticism and conscientiousness on sleep quality is an important perspective to consider. Additional research of this nature could provide insight into potential mechanisms involved in the relationship between personality and sleep behaviours.

In addition to sleep behaviours, personality has also been linked to depression. Individuals with depression often exhibit higher levels of neuroticism and lower levels of extraversion, openness, and conscientiousness [31]. Neuroticism is considered to be a substantial risk factor involved in the etiology of depression [32], in both clinical [33, 34] and non-clinical samples [35, 36]. Several longitudinal studies using large community samples have identified higher levels of neuroticism to be a unique predictor of the onset of an initial major depressive episode [37–40]. Higher neuroticism is also associated with greater depressive symptom severity [31].

While high levels of neuroticism are strongly associated with depressive symptoms, numerous studies have demonstrated that conscientiousness is also a predictor of depression such that major depression is associated with high levels of neuroticism and low levels of conscientiousness [41, 42]. A longitudinal study by Roberts, Caspi and Moffitt [43] found that constraint at age 18, a specific facet of conscientiousness, was positively predictive of important work-related outcome variables such as work satisfaction and financial security at age 26. Furthermore, in a 30-year longitudinal study, social responsibility, another facet of conscientiousness, assessed at age 21, was negatively associated with factors of divorce, and tobacco use, at midlife and final assessment ages (43 and 52 years) in a large sample of women [44]. Generally, conscientiousness can be a protective personality trait, whereas neuroticism can render an individual more vulnerable to developing/experiencing depressive symptoms. Examining personality traits may serve as a method to identify individuals who might be at risk for developing depression and help to inform treatment [45]. The relationship between personality and depression however is complex, and a more comprehensive approach is necessary in order to examine other contributing factors or mechanisms in this relationship.

While studies have identified personality and sleep as important risk factors for depression, these studies have taken a simplistic approach by examining each association in isolation (i.e. the association between personality and depression or the association between sleep and depression) and have mostly focused on young adult cohorts. To date, there is a paucity of research investigating the potential mediating role of subjective sleep quality in the interplay between personality traits and depressive symptoms in middle-aged adults.

Given the well-documented association between personality traits and depression, as well as personality traits and sleep, it is plausible that personality traits may indirectly impact depression through subjective sleep quality. Thus, the purpose of this study was to examine the relationship between the Five Factor personality traits, depressive symptoms, and subjective sleep quality in middle-aged adults. Based on previous literature, it is hypothesized that neuroticism will be positively associated with self-reported sleep disturbance and depressive symptomology, whereas conscientiousness will have negative relationships with subjective sleep quality and depressive symptoms. Furthermore, this is the first study to employ a mediation model to examine the mediating role of subjective sleep quality in the relationship between personality traits (i.e., neuroticism and conscientiousness, examined separately) and depressive symptoms in a group of middle-aged adults.

## Method

### Participants

A total of 114 middle-aged adults (*M* = 57.92 years, *SD* = 4.00, range = 47–67 years) were recruited from the community. Participants were excluded if they reported past or current history of neurological, psychiatric, and/or other medical conditions (e.g., diabetes) that could affect cognitive function. The majority of the sample identified as Caucasian (88.6%), 76.6% were females, 48.2% reported university-level education and 40.0% were retired (see Table 1). The study was approved by the Douglas Mental Health University Institute (REB#03/40). Written informed consent was obtained from all participants prior to their participation.

**Table 1:** Sample Characteristics and Descriptive Statistics of Continuous Variables.

	Total (*N* = 114)
Mean age, years (*SD*)	57.92 (4.01), range: 47–67
Gender (Female %)	76.3
Ethnicity (%)	
Caucasian	88.6
African American	3.5
Indian	2.6
Spanish	5.3
Native Language (%)	
English	69.3
French	23.7
Other—tested in English	4.4
Other—tested French	2.6
Education, years (*SD*)	16.00 (3.30)
Elementary (%)	1.8
High School/ CEGEP (%)	38.6
Technical School (%)	12.3
University	48.2
Marital status (%)	
Single	27.2
Married	35.1
Divorced/Separated	34.2
Widowed	3.5
Employment status (%)	
Full-time	10.9
Part-time	34.5
Still looking	7.3
Self-employed	7.3
Retired	40.0
Self-perceived socio-economic status (%)	
Low	43.7
Medium	43.7
High	12.6
Medication (%)	
None	71.1
Blood pressure/cholesterol	18.4
Varicose veins	0.9
Glaucoma drops	2.6
Osteoporosis	2.6
Arthritis	1.8
Stomach/acid reflux	1.8
Sleep aids	0.9
CES-D *(SD*)	9.68 (9.33)
Neuroticism (*SD*)	16.81 (8.62)
Conscientiousness (*SD*)	34.99 (6.71)
Subjective sleep quality (*SD*)	0.91 (0.86)
Sleep latency (*SD*)	0.89 (0.89)
Sleep duration (*SD*)	0.57 (0.86)
Sleep efficiency ^*a*^ (*SD*)	0.54 (0.91)
Sleep disturbances (*SD*)	1.39 (0.54)
Use of sleep medication (*SD*)	0.20 (0.63)
Daytime dysfunction (*SD*)	0.77 (0.68)
Global PSQI score (*SD*)	5.28 (3.45)
Sleep apnea symptoms (*%*)	58.5 (*n* = 41)
Restless leg syndrome (%)	36.6 (*n* = 41)

*Notes*. CEGEP = General and vocational college; CES-D = Centre for Epidemiologic Studies Depression Scale; PSQI = Pittsburgh Sleep Quality Index.

a—this score reflected the PSQI sleep efficiency component score.

### Measures

#### The Center for Epidemiologic Studies Depression Scale

The Center for Epidemiologic Studies Depression Scale (CES-D; [46]) is a 20-item self-report measure of depressive symptoms. The scale has been validated and used in community settings [26, 47]. All items are rated on a 4-point Likert-type frequency scale, ranging from 0 (*Rarely or none of the time*) to 3 (*Most or all of the time*). The total score on the CES-D ranges from 0 to 60, with higher scores reflecting greater depressive symptom severity. A cut-off score of 16 is recommended to define a clinically significant level of depressive symptoms [46, 48]. The CES-D demonstrated good internal consistency in the current sample (*α* = .91).

#### The Pittsburgh Sleep Quality Index

The Pittsburgh Sleep Quality Index (PSQI; [49]) is a 19-item self-report measure that assesses subjective sleep quality and sleep disturbances over a 1-month time period. The first four items are open-ended format (e.g., regular bedtime), whereas items 5 to 19 are reported on a 4-point Likert-type frequency scale, ranging from 0 (*Not during the past month*) to 3 (*three or more times a week*). The scale is also comprised of seven sleep components through various algorithms: subjective sleep quality, sleep latency, sleep duration, habitual sleep efficiency, sleep disturbances, use of sleep medications, and daytime dysfunction. The global PSQI score ranges from 0 to 21, which is obtained by adding the seven component scores. In addition, there are five items to measure possible sleep apnea and restless leg syndrome (RLS) symptoms by their roommate or bed partners. A global score greater than five is an indication of sleep impairment. The scale exhibits good psychometric properties, with internal consistency ranging from 0.80 to 0.83 [49], good test-retest reliability [50], and convergent validity with other sleep measures [51] and sleep-log [49]. The scale demonstrated adequate internal consistency in the present community-dwelling sample (*α* = .75).

#### The Short Form NEO Five Factory Inventory

The short form NEO Five Factory Inventory (NEO-FFI; [21]) is a self-report measure that assesses each of the five major personality traits including neuroticism, extraversion, openness, agreeableness, and conscientiousness. The scale is comprised of 60 items. Each personality trait is measured in 12 items. All items are rated on a 5-point Likert-type scale, ranging from 0 (*strongly disagree*) to 4 (*strongly agree*). The total score on the NEO-FFI ranges from 0 to 48, with higher scores reflecting greater endorsement of the specific trait. The NEO-FFI has demonstrated good internal consistency [21]. The NEO-FFI demonstrated adequate to good internal consistency across the five subscales in the current sample (*α* = 0.76–0.86). For the purpose of the current study, only neuroticism and conscientiousness traits were examined.

### Statistical Analysis

All analyses were performed using SPSS v.22 [52]. An alpha level of 0.05 was used to determine statistically significant associations. Descriptive and correlational analyses were conducted to assess sample characteristics and the relationships between variables.

**Fig 1:**
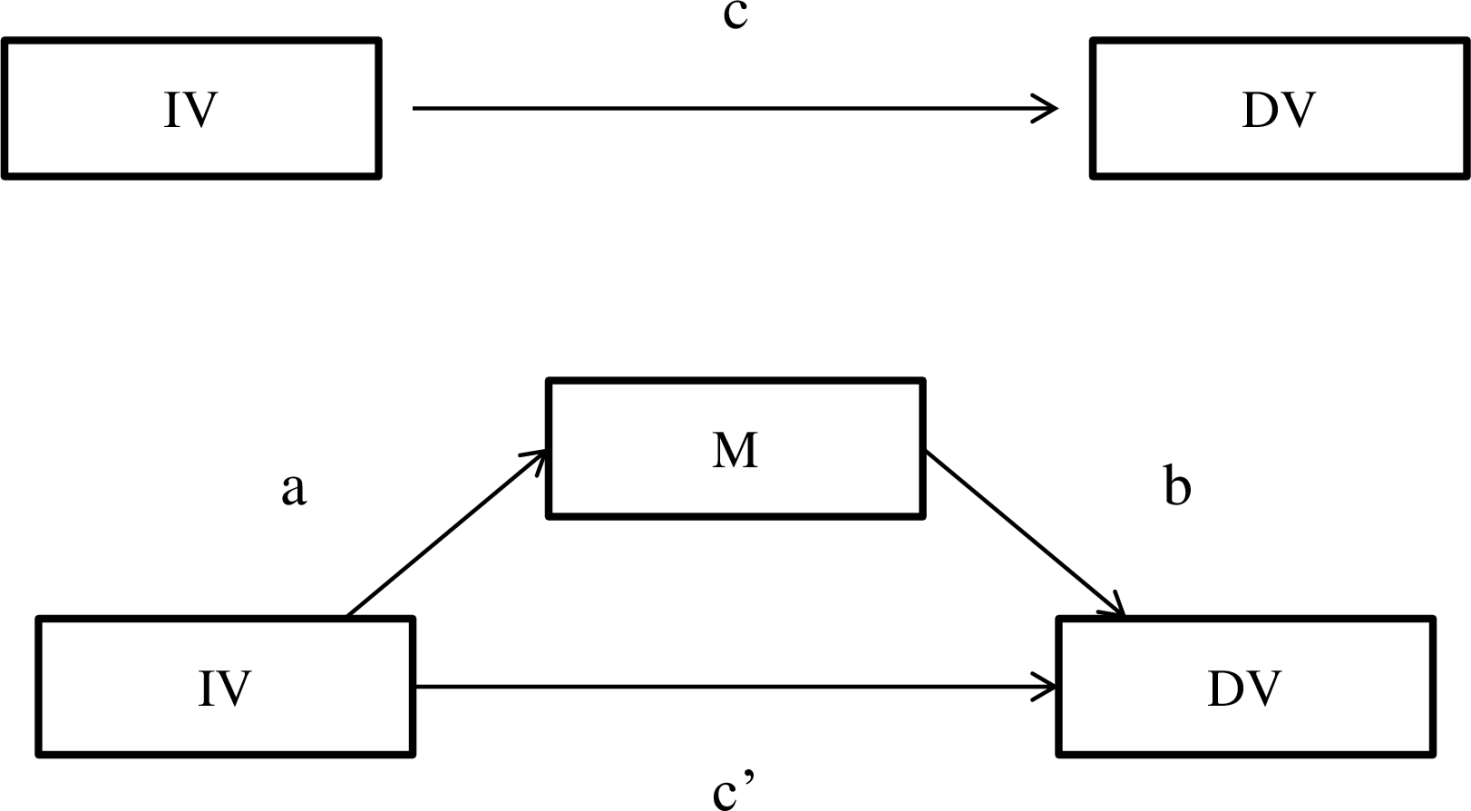
Mediation model. IV = Independent variable; DV = dependent variable; M = Mediator; a = direct relationship between IV and M; b = direct relationship between DV and M; c = direct relationship between IV and DV; c’ = Indirect mediation effect of IV and DV through M.

A mediational analysis [53] was conducted to determine how sleep might influence the association between personality traits and depressive symptoms. Previous research has established that high neuroticism and low conscientiousness are associated with greater experience of depressive symptoms [42]. Thus, the current study employed neuroticism and conscientiousness as the independent variables and depressive symptoms as the outcome variable. Given that age, sex, and medication have been shown to influence sleep and depressive symptoms, these variables were entered as covariates in the mediation analyses [54]. As indicated in the hypotheses, self-report sleep quality was entered in the model as the mediator that influences the relationship between personality traits and depressive symptoms. Fig 1 depicts the simple mediation model between personality traits, sleep, and depressive symptoms.

The PROCESS macro on SPSS developed by Hayes [55] was used to test the mediation model. The macro tests mediation models by comparing the observed indirect effect against 5000 bootstrapped resamples [53]. Each simulated parallel dataset is constructed by random sampling from the observed dataset with replacement. Based on the distribution of the resampled datasets, 95% confidence intervals (CI) were generated for the different effects being examined (i.e., the total, direct, and indirect effects). If the CI does not include zero, the indirect mediation is significantly different than zero [55, 56]. The bootstrapping method was utilized, as it is a more recent and highly recommended method to examine indirect mediation effects [53, 57, 58].

Researchers have also suggested examining other equivalent mediation models to rule out possible statistical equivalency [59, 60]. Although a theoretical background has been established for the current hypothesized model, an alternative mediation analyses was computed to rule out alternative models. The alternative explorative model entered depressive symptoms as the mediator, personality traits as the independent variable and sleep as the outcome variable.

## Results

### Descriptive and Correlational Analysis

Table 1 provides sample characteristics and descriptive statistics for all measures. Overall, the current sample reported low sleep impairment with a mean of the global PSQI scores of 5.28 (*SD* = 3.45). Twenty-four participants reported symptoms of sleep apnea and 15 participants reported restless leg syndrome (RLS) by their roommate or bed partner. The sample reported low depressive symptoms as reflected in the mean CES-D score, which is lower than the clinical cut-off of 16. The current sample scored low on neuroticism and high on conscientiousness traits.

Pearson’s correlation was conducted to examine the associations between demographic variables, personality traits, sleep variables, and depressive symptoms (see Table 2). Variance inflation factors and tolerance levels indicated that the assumption of multi-collinearity was not violated. Consistent with hypothesis 1, Neuroticism was positively correlated with CES-D scores and self-report sleep quality through the PSQI components and the global PSQI score. Neuroticism was not correlated with sleep efficiency, sleep duration, and daytime dysfunction components (see Table 2). Conscientiousness was negatively correlated with depressive symptoms, sleep efficiency and daytime dysfunctions (see Table 2). Depressive symptoms were significantly correlated with all PSQI components and global PSQI score, except for medication used (see Table 2). Subsequent mediation analyses were analyzed for variables that displayed significant correlations.

**Table 2:** Correlations between Demographic Variables and Continuous Measures (N = 114).

	1	2	3	4	5	6	7	8	9	10	11	12
Age (1)												
EDU (2)	.02											
CES-D (3)	-.07	-.01										
N (4)	-.13	-.04	.63**									
C (5)	-.03	.09	-.39***	-.37***								
SE (6)	.06	-.21*	.27**	-.17	.06							
SL (7)	-.05	-.15	.45***	.46***	-.19*	.41***						
SDur (8)	.04	-.11	.21*	.14	.01	.55***	.30**					
SDis (9)	.05	-.10	.34***	.28**	-.13	.43***	.40***	.29**				
SQ (10)	-.12	-.14	.39***	.29**	-.01	.47***	.55***	.46***	.53***			
Med (11)	.07	.02	.08	.20*	-.07	.03	.17	.08	.18	.15		
DDys (12)	.08	.03	.35***	.17	-.32**	.13	.20*	.21*	.42***	.21*	.11	
PSQI (13)	.02	-.17	.47***	.38***	-.13	.72***	.71***	.69***	.69***	.78***	.34***	.47***

*Notes*. EDU = Years of Education; CES-D = Center for Epidemiologic Studies Depression Scale; N = Neuroticism; E = Extraversion; O = Openness; A = Agreeableness; C = Conscientiousness; SE = Sleep efficiency; SL = Sleep latency; SDur = Sleep duration; SDis = Sleep disturbance; SQ = Subjective sleep quality; Med = Use of sleep medication; DDys = Daytime Dysfunction; PSQI = PSQI global score.

* *p* < .05

** *p* < .01

*** *p* < .001.

### The Relationship between Neuroticism and Depressive Symptoms: Sleep as the Mediator

#### Sleep latency

Adjusting for age, gender, and medication use, bootstrap indirect mediation analysis revealed a partial mediation in the relationship between neuroticism and depressive symptoms through sleep latency. A small, significant indirect effect was found in this mediation model (.11, 95% CI (.02, .22)). Together, neuroticism and sleep latency account for 44% of the variance in depressive symptoms (*F*(5, 108) = 16.75, *p* < .001).

#### Sleep disturbance

Adjusting for age, gender, and medication, bootstrap indirect mediation analysis revealed a small, partial indirect effect of sleep disturbance in the relationship between neuroticism and depressive symptoms (.06, 95% CI (.01, .13)). Together, neuroticism and sleep disturbance accounts for 42% of the variance in depressive symptoms, controlling for age, gender, and medication (*F*(5, 108) = 15.77, *p* < .001).

#### Sleep quality

Adjusting for age, gender, and medication, bootstrap indirect mediation analysis revealed a small, partial indirect effect of sleep quality in the association between neuroticism and depressive symptoms (.07, 95% CI (.01, .18)). Neuroticism and sleep quality accounted for 45% of the variance in CES-D scores (*F*(5, 108) = 17.48, *p* < .001).

#### Sleep medication

There was no significant indirect effect of medication use in the relationship between neuroticism and depressive symptom, controlling for age, gender, and medication (-.01, 95% CI (-.06, .03)).

#### Global PSQI

The bootstrap method supported a small, partial indirect effect of global PSQI score in the relationship between neuroticism and depressive symptom (.12, 95% CI (.05, .22)). Adjusting for both age, gender, and medication, both neuroticism and global PSQI scores accounted for 46% of the variance in depressive symptoms (*F*(5, 108) = 18.62, *p* < .001).

#### Combined sleep components

A final bootstrap mediation analysis was conducted with all aforementioned sleep components entered as mediators, along with neuroticism as the predictor and depressive symptom as the outcome variable. With the combined sleep components as the mediator, this model partially mediated the relationship between neuroticism and depressive symptoms (.11, 95% CI (.01, .23)). Together, neuroticism with all sleep components accounted for 48% of the variance in depressive symptoms (*F*(9, 104) = 10.71, *p* < .001). Overall, the hypothesis that self-report sleep measures serve as a mediator in the relationship between neuroticism and depressive symptoms was partially supported. See Table 3.

**Table 3:** Significant Indirect Mediation Analysis of Neuroticism, PSQI, and CES-D.

Model		β	*p*
Hypothesized model			
N → Sleep latency → Depressive symptoms	N → Sleep latency	0.04	.000
	Sleep latency → Depressive symptoms	2.45	.006
	N → Depressive symptoms	0.58	.000
N → Sleep disturbance → Depressive symptoms	N → Sleep disturbance	0.02	.001
	Sleep disturbance → Depressive symptoms	2.97	.029
	N → Depressive symptoms	0.63	.000
N → Sleep quality → Depressive symptoms	N → Sleep quality	0.03	.006
	Sleep quality → Depressive symptoms	2.63	.002
	N → Depressive symptoms	0.62	.000
N → Global PSQI → Depressive symptoms	N → Global PSQI	0.15	.006
	Global PSQI → Depressive symptoms	0.77	.000
	N → Depressive symptoms	0.57	.000
Alternative Model			
N → Depressive symptoms → Sleep latency	N → Depressive symptoms	0.69	.000
	Depressive symptoms → Sleep latency	0.03	.006
	N → Sleep latency	0.03	.022
N → Depressive symptoms → Sleep disturbance	N → Depressive symptoms	0.69	.000
	Depressive symptoms →→ Sleep disturbance	0.02	.029
	N → Sleep disturbance	0.01	.225
N → Depressive symptoms → Sleep quality	N → Depressive symptoms	0.69	.000
	Depressive symptoms → Sleep quality	0.03	.002
	N → Sleep quality	0.003	.758
N → Depressive symptoms → Global PSQI	N → Depressive symptoms	0.69	.000
	Depressive symptoms → Global PSQI	0.15	.000
	N → Global PSQI	0.05	.250

*Notes*. Mediation analyses were conducted on PROCESS macro, adjusting for age, gender and medication. N = neuroticism; PSQI = Pittsburgh Sleep Quality Index; CES-D = Centre for Epidemiologic Studies-Depression Scale.

### The Relationship between Conscientiousness and Depressive Symptoms: Sleep as the Mediator

#### Sleep latency

Adjusting for age, sex, and medication, bootstrap indirect mediation analysis revealed a small, partial indirect effect of latency (-.12, 95% CI (-.25, -.03)) in the relationship between conscientiousness and depressive symptoms. Together, conscientiousness and sleep latency accounted for 31% of the variance in depressive symptoms (*F*(5, 108) = 9.55, *p* < .001).

#### Daytime dysfunction

Adjusting for age, gender, and medication, bootstrap indirect mediation analysis revealed daytime dysfunction component partially mediated the relationship between conscientiousness and depressive symptoms (-.11, 95% CI (-.26, -.04)). Both conscientiousness and daytime dysfunction accounted for 23% of the variance in depressive symptoms (*F*(5, 108) = 6.32, *p* < .001).

#### Combined sleep components

The combined sleep component model significantly mediated the relationship between conscientiousness and depressive symptoms (-.20, 95% CI (-.35, -.08)). Together, sleep latency, daytime dysfunction and conscientiousness accounted for 34% of the variance in depressive symptoms (*F*(6, 107) = 9.20, *p* < .001). Overall, the hypothesis that self-report sleep measures mediate the relationship between conscientiousness and depressive symptoms was partially supported (See Table 4).

**Table 4:** Significant Indirect Mediation Analysis of Conscientiousness, PSQI, and CES-D.

Model		β	*p*
Hypothesized model			
C → Sleep latency → Depressive symptoms	C → Sleep latency	-0.03	.019
	Sleep latency → Depressive symptoms	4.20	.000
	C → Depressive symptoms	-0.44	.000
C → Daytime dysfunction → Depressive symptoms	C → Daytime dysfunction	-0.03	.001
	Daytime dysfunction → Depressive symptoms	3.61	.004
	C → Depressive symptoms	-0.44	.001
Alternative Model			
C → Depressive symptoms → Sleep latency	C → Depressive symptoms	-0.56	.000
	Depressive symptoms → Sleep latency	0.04	.000
	C → Sleep latency	-0.006	.608
C → Depressive symptoms → Daytime dysfunction	C → Depressive symptoms	-0.56	.000
	Depressive symptoms → Daytime dysfunction	0.02	.004
	C → Daytime dysfunction	-0.02	.036

*Notes*. Mediation analyses were conducted via PROCESS macro, adjusting for age, gender and medication. C = conscientiousness; CES-D = Centre for Epidemiologic Studies Depression Scale; PSQI = Pittsburgh Sleep Quality Index.

### Alternative Explorative Model: Depression as the Mediator in the Relationship Between Personality and Sleep

#### Neuroticism

A significant indirect effect of depressive symptoms was found for the relationship between neuroticism and sleep latency (.02, 95% CI (.003, .03)), accounting for 30% of the variance in sleep latency component scores (*F*(5, 108) = 9.15, *p* < .001). A small indirect effect of depressive symptoms was fund for the relationship between neuroticism and sleep disturbance (.01, 95% CI (.002, .02)), accounting for 17% of the variance in the sleep disturbance component (*F*(5, 108) = 4.25, *p* = .001). Depressive symptoms completely mediated the relationship between neuroticism and sleep quality component (.02, 95% CI (.01, .04)), accounting for approximately 20% of the variance in sleep quality (*F*(5, 108) = 5.32, *p* < .001). There was no evidence of mediation in the alternative model with depressive symptom as the mediator in the relationship between Neuroticism and sleep medication (-.004, 95% CI (-.02, .008)). Finally, depressive symptoms completely mediated the relationship between neuroticism and global PSQI scores (.01, 95% CI (.04, .17), accounting for 26% of the variance in global PSQI scores (*F*(5, 108) = 7.64, *p* < .001).

Although the alternative models suggest that depressive symptoms serve as a mediator in the relationship between neuroticism and self-report sleep measures, the hypothesized model (i.e. sleep as mediator) accounted for greater portion of the variance in depressive symptoms along with neuroticism in comparison to the alternative model. See Table 3.

#### Conscientiousness

To explore the alternative model, mediation analysis resulted in a complete indirect effect of depressive symptom in the relationship between conscientiousness and sleep latency (-.02, 95% CI (-.04, -.01)), accounting for 26% of the variance in the sleep latency component scores. Further, a small, partial indirect effect of depressive symptoms in the relationship between conscientiousness and daytime dysfunction was found (-.01, 95% CI (-.02, -.004)), accounting for 18% of the variance in daytime dysfunction (*F*(5, 108) = 4.87, *p* < .001).

Again, the alternative model suggests that depressive symptoms may also mediate the relationship between conscientiousness and sleep measures. However, the hypothesized model (i.e., sleep as the mediator) accounted for a greater portion of the variance in depressive symptoms. See Table 4.

## Discussion

Depressive symptoms play a crucial role in the physical, cognitive, and emotional well-being in middle-age adults. Individual differences, such as personality traits, have been identified as risk factors for the onset of depression. In addition to personality traits, sleep has received increasing attention for its role in the etiology of depression. Although studies have examined the relationship between personality traits and sleep or personality as a correlate of depression, few studies have explored these constructs in a single model. The current study sought to examine the mediating role of subjective sleep quality in the relationship between personality traits and depression. Given that this is one of the few studies that examine the aforementioned relationship, the present study took a more exploratory approach in investigating this mediation model and its alternate.

As expected, neuroticism was positively associated with depressive symptoms and subjective sleep quality, whereas conscientiousness was negatively related to depressive symptoms and subjective sleep quality in a group of community-dwelling middle-aged adults. Consistent with previous research, high neuroticism was linked to greater experience of depressive symptoms and sleep issues, whereas high conscientiousness was associated with lower depressive symptoms and better sleep [41, 61, 62, 63]. Moreover, significant relationships were found between subjective sleep quality and depressive symptoms, as supported by previous findings [64, 65].

The relationship between neuroticism and depressive symptoms were partially mediated by both the unique PSQI components (i.e., self-report sleep latency, sleep disturbance, and sleep quality), as well as the global PSQI scores. When the aforementioned components are combined, the total model also partially mediated the relationship, after controlling for age, gender, and medication use. Moreover, the combined model accounted for almost half of the variance in depressive symptoms. This suggests that the relationship between neuroticism and depression is significantly explained by subjective sleep quality, a relationship that is enhanced with the presence of a combination of sleep indices. Previous studies suggest that neuroticism is related to stress sensitivity [66], which is a predisposing factor for insomnia [67], as well as for depression [68, 69]. With increased vulnerability to daily stressors, individuals with high neuroticism may experience poor sleep quality and greater sleep disturbance due to high levels of stress. It is suggested that stress is linked to greater sleep onset latency and disruption of the sleep cycles [70, 71]. When accompanied by maladaptive coping strategies due to sleep-related cognitive impairment (e.g., inability to concentrate), high neuroticism may further contribute to an increase in negative mood (e.g., dysphoria).

The alternative model revealed that depressive symptoms fully mediate the relationship between neuroticism and different sleep components. Previous studies have consistently identified neuroticism as a risk factor for depression [32, 72]. As a hallmark symptom of depression, sleep disturbance is often experience in individuals with depression [73, 74]. Thus, the current study provided preliminary support that depressive symptoms mediate the relationship between neuroticism and subjective sleep quality. At first glance, the alternative model may explain the relationship between personality traits, sleep, and depressive symptom better compared to the hypothesized model. However, the alternative model only accounted for a small portion of the variance, after controlling for the *a priori* covariates. Therefore, it is plausible that there are other factors that have not been accounted for in the current model. Given the complex relationship between sleep and depression, future studies should explore other potential moderators or mediators in the personality-sleep-depression interplay.

A partial indirect mediating effect was found in the relationship between conscientiousness and depressive symptoms through self-report sleep latency component and daytime dysfunction. By entering both sleep components as the mediator, the association between conscientiousness and depressive symptoms remained partially mediated through the two sleep components. The current findings suggest that low conscientious individuals are likely to experience depressive symptoms through taking a longer period of time to fall asleep and greater daytime dysfunction. Although limited studies have examined the link between conscientiousness and sleep, previous findings suggest that high conscientiousness is associated with good sleep quality and less daytime dysfunction in young adults [25, 29, 75]. Moreover, previous studies suggest that high conscientiousness is negatively associated with depression and treatment outcome [31, 76]. Given that conscientiousness is characterized by being responsible and organized, high conscientiousness has been associated with better health outcome and good coping skills [77, 78]. Thus, the link between conscientiousness and depressive symptoms may be mediated through the use of adaptive coping strategies to manage daily stressors and engaging in health-promoting behaviours, such as practicing good sleep hygiene [30, 79, 80].

Similar to neuroticism, the alternative model revealed that depressive symptoms completely mediate the relationship between conscientiousness and sleep latency, whereas depressive symptoms only partially mediate the association between conscientiousness and daytime dysfunction. Given that sleep disturbance is one of the symptoms of depression [81], the presence of depressive symptoms may attenuate the protective nature of conscientiousness on subjective sleep quality. Thus, the current study provides preliminary evidence for an indirect effect of depressive symptoms in the relationship between conscientiousness and subjective sleep quality. Similar to findings of neuroticism, the alternative model of conscientiousness only accounted for a small portion of the variance in sleep components. It is plausible that additional moderators or mediators may also be present in the current model, especially within the sleep-depressive symptoms link.

**Fig 2:**
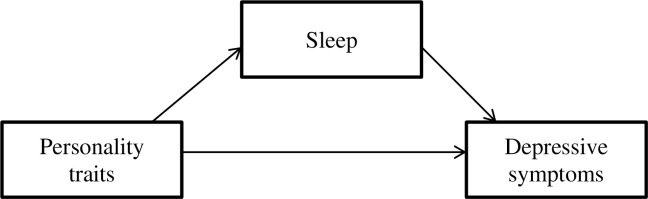
Model 1: Sleep partially mediates the relationship between personality traits and depressive symptoms.

**Fig 3:**
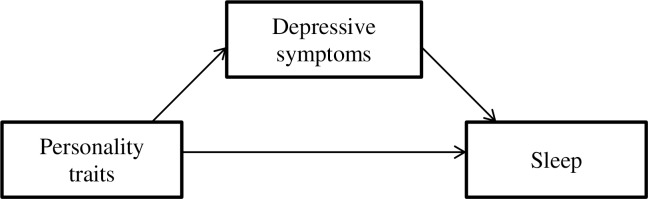
Model 2: Depressive symptoms fully mediated the relationship between personality traits and sleep.

Taken together, two models can be constructed (Figs 2 and 3). The current findings yield small effect sizes for the mediating role of subjective sleep quality in relation to personality traits and depressive symptoms. Despite these small effect sizes, the examination of sleep in the personality-depression context may enhance knowledge in understanding the mechanisms that connect personality traits, sleep, and depression, especially in middle adulthood. Although predisposed individual differences may serve as a risk factor for depression, depressive symptoms are not manifested in all individuals who score high on a specific personality trait. It appears that subjective sleep quality and disturbance, in combination with personality traits, might provide a gateway to identifying individuals who may be vulnerable to experiencing depressive symptoms. It may also be suggested that depressive symptoms reduce the influence of personality traits on sleep.

Knowledge gained from the current findings may aid in the creation of personalized intervention strategies for individuals with sleep problems and depression. For instance, personality trait assessments can be implemented in clinics to help clinicians develop an appropriate treatment plan for those reporting higher levels of neuroticism. Furthermore, psychoeducation programs (e.g., stress reduction, social engagement, and sleep hygiene) may benefit individuals with different personality profiles.

### Strengths, Limitations, and Future Research Directions

The current study was the first to examine the mediating role of self-report sleep in the relationship between personality traits and depressive symptoms in a group of middle-aged adults. This was also the first study to use bootstrapping indirect mediation analyses to examine the relationship between the three variables. All variables were assessed using well-validated measures. Moreover, the present study provided a comprehensive evaluation of personality, and sleep by using the Five Factor model and all components of the PSQI to assess different aspects of sleep.

The present findings should be considered in light of the study’s limitations. While this research supports other studies linking sleep disturbance and personality traits with depression, it shares certain methodological weaknesses consistent with the field as a whole. Most importantly, a cross-sectional design was used in this study. Although there are sound theoretical reasons for treating our covariates (i.e., age, sex, medication) as potential determinants of sleep disturbance, our cross-sectional data prevent the identification of causal relations. Additionally, since we cannot temporally separate the development of personality traits, sleep, and depression in this sample, we cannot definitively rule out alternative explanations to the observed associations. For instance, we did not assess or control for potential confounders such as previous history of depression or insomnia, coping strategies, existing stressors, caffeine consumption, or physical activity, which are associated with depression onset and sleep disturbance [10, 82, 83, 84].

Another threat to causal interpretation arises from the possibility that personality traits (e.g., neuroticism) may affect the way in which individuals perceive their sleep quality and mood, leading them to report their symptoms as more severe. Thus, objective measures of sleep, personality, and depressive symptoms would be preferable. Moreover, neuroticism is associated with the frequent experience of negative affect [21]. Given that neuroticism and depression share similar underlying characteristics and are both measured subjectively, it is somewhat difficult to disentangle their relationship over time. However, this argument does not extend to the personality trait conscientiousness, which does not share features with depression. Finally, the current study used a non-representative sample. The majority of the sample consisted of females who self-identified as Caucasians, and were highly educated. As a result, it is difficult to generalize the current findings to a more diverse sample.

Future investigations should assess a broader set of relevant covariates within a larger community sample of middle-aged adults. Although the PSQI is well validated, it only assesses the average of all sleep variables within the past month. Sleep latency, sleep duration, and sleep efficiency were assessed using four estimated times (i.e., usual bed time, number of minutes to fall asleep, usual wake time, and number of hours of sleep per night in the past month), combined. These broad estimates are not sufficient to capture the daily variance of these important sleep components. Thus, the broad estimated time points might not reflect the accurate and precise sleep components. Future studies should use a sleep log, such as the consensus sleep diary [85], and objective sleep measures (e.g., actigraph or polysomnography) to monitor participants’ daily sleep-wake cycle. More specifically, the use of polysomnography could monitor the neural activity and provide a precise measure for the length of each stage of sleep. Furthermore, information obtained from actigraph or polysmonography could compensate for the over- or under-estimation of the amount of time spent being awake and sleeping in subjective measures. Such methods will provide more accurate measures of the aforementioned sleep variables. Longitudinal research is needed to further examine the interplay between individual variances, sleep and depression, as well as to validate the current mediation model.

## Conclusion

With a growing aging population and late onset depression becoming more common, it is imperative to explore the effect of individual differences on the development of depression and the factors that may mediate or moderate this relationship. Despite the aforementioned limitations, the current study provides preliminary findings for a novel pathway to understand the personality-sleep-depression relationship. Particularly, subjective sleep quality had an indirect mediating effect on the relationship between personality traits and depressive symptoms in a group of community-dwelling middle-aged adults. Future studies should replicate the current study using longitudinal design and explore other individual variables (e.g., genetic variances) that may underlie the association between sleep and depression.

## Supporting Information

S1 File Dataset. Minimal dataset associated with this article.
(SAV)
